# A laser‐assisted endoscopic approach to pyriform sinus fistula via fibrin glue cauterization

**DOI:** 10.1002/ccr3.6588

**Published:** 2022-12-12

**Authors:** Manami Momii, Toshiaki Kawano, Sonoka Takakura, Takashi Hirano, Kaori Tateyama, Masashi Suzuki

**Affiliations:** ^1^ Department of Otolaryngology & Head and Neck Surgery, Faculty of Medicine Oita University Oita Japan

**Keywords:** endoscopic ablation, fibrin glue, laser cauterization, pyriform sinus fistula

## Abstract

In the past, the general treatment method for pyriform sinus fistula was its removal by open surgery; however, in recent years, endoscopic surgery has become more common. We report two cases where laser surgery was performed using an endoscope and recurrence was prevented using fibrin glue. Both cases involved 3‐year‐old girls who underwent laser ablation of a pyriform sinus fistula under an endoscope, after which the site was closed with fibrin glue. No recurrence was observed in either case, and the postoperative course was uneventful. This approach is presented as a non‐invasive and effective treatment for pyriform sinus fistula.

## Abstract 2

This paper reports the cases of endoscopic laser ablation with fibrin glue application for pyriform sinus fistula (PSF) closure. This makes a significant contribution to the literature because recurrence of PSF was prevented using fibrin glue.

## 1 INTRODUCTION

Pyriform sinus fistula (PSF) causes repeated cervical abscesses and acute purulent thyroiditis in young people.^1^ Surgical treatment is recommended as a curative treatment for PSF.^2^ In the past, fistula removal by extra cervical incision was common, but recent reports of cauterization of the fistula opening using endoscopy have been accepted due to aesthetic factors.^3^ Furthermore, surgery aimed at improving the recurrence rate has been performed by closing the PSF with fibrin glue.^4^ We report two cases of children where PSF was closed using fibrin glue under laser assistance at our facility.

## 2 CASE REPORTS

**FIGURE 1:**
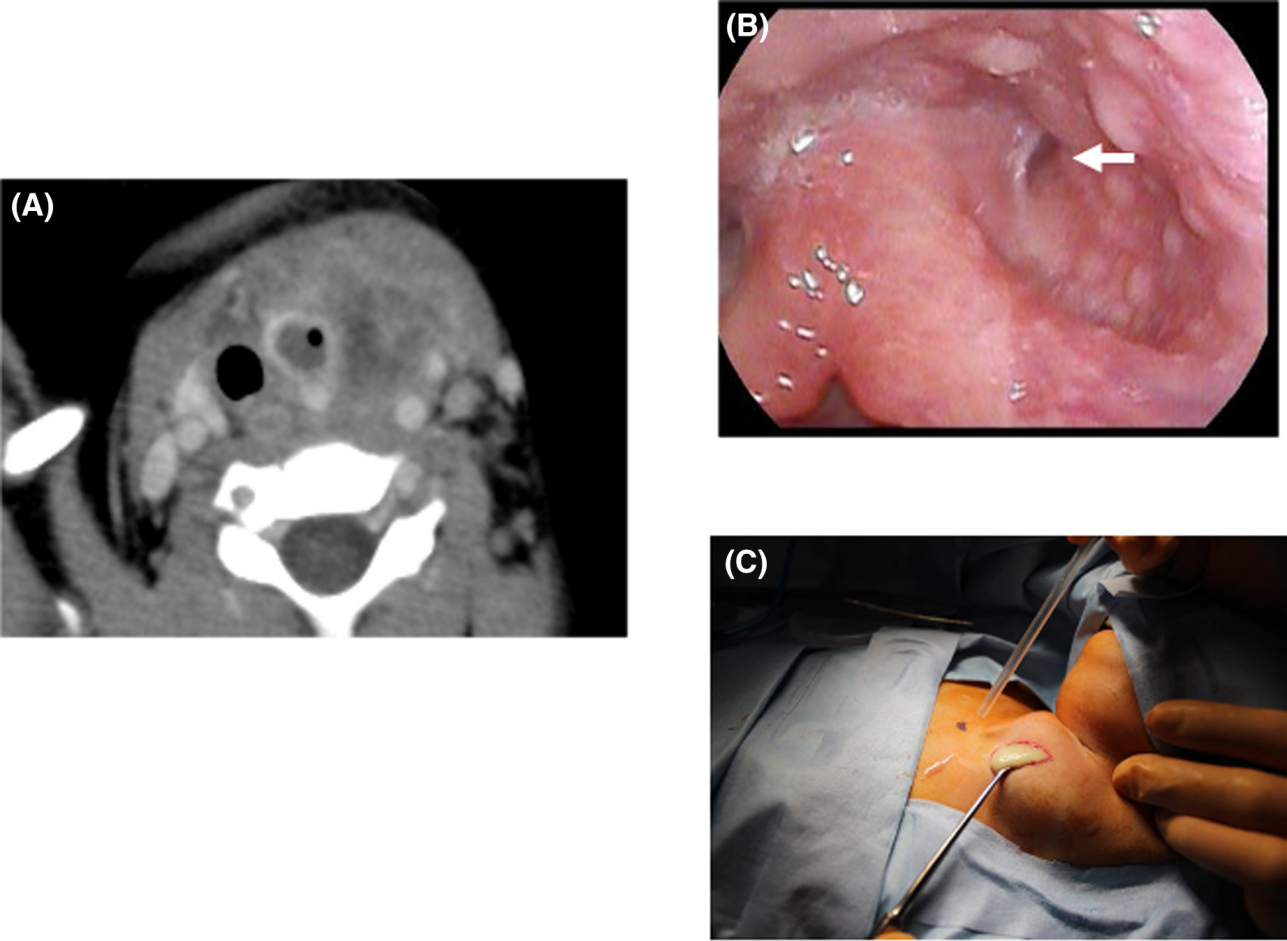
(A) Contrast‐enhanced computed tomography shows a low absorption area including ring enhancement so that it partially penetrates the left pyriform sinus and the left lobe of the thyroid. (B) A fistula opening was confirmed in the left pyriform sinus by throat endoscopy (white arrowhead). (C) Under general anesthesia, left cervical abscess incision and drainage were performed. A large amount of pus was drained, the inside was washed, and a drain was placed

**FIGURE 2:**
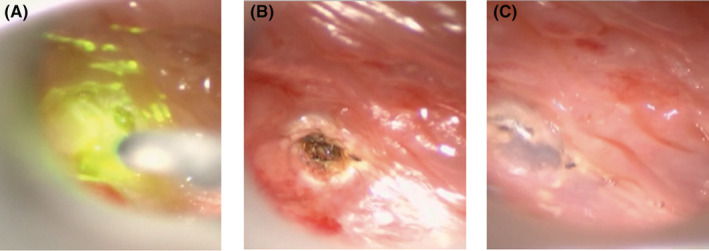
(A) After inserting the laryngeal endoscope, a fistula opening was confirmed in the left pyriform sinus, and the site was completely cauterized with a diode laser. (B) It was confirmed that the fistula opening was sufficiently cauterized. (C) Fibrin glue was injected into the fistula cautery, and it was confirmed that the fistula was closed

**FIGURE 3:**
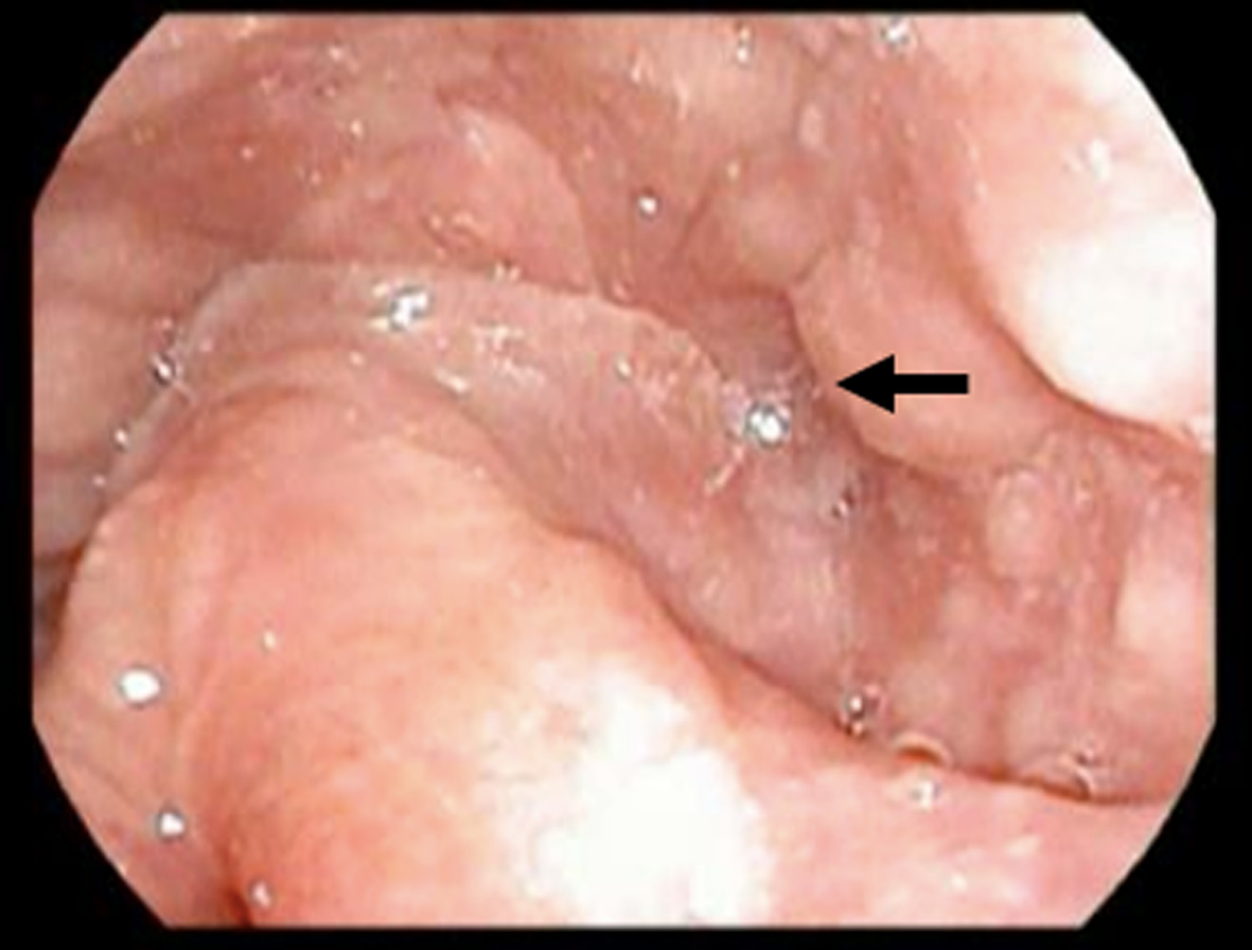
Pharyngeal endoscopic findings 8 months after the operation confirmed that there was no recurrence at the fistula closure site

Case 1: A 3‐year‐old girl presented with a fever and left posterior cervical swelling. After visiting a nearby hospital, she was referred to our department because of a cervical abscess on contrast‐enhanced computed tomography (CT). After visiting our department, she underwent cervical abscess puncture and drainage under local anesthesia. Her infection resolved; however, she developed a fever and left cervical swelling again. Contrast‐enhanced CT showed a low absorption area on the back of the left lobe of the thyroid gland and a cervical abscess (Figure 1A). A continuous abscess cavity was confirmed in the left pyriform sinus (Figure 1B), and cervical abscess incision and drainage were performed under general anesthesia (Figure 1C). After her second infection subsided, she underwent oral endoscopic pyriform sinus ablation under general anesthesia. Observation with an endoscope revealed a fistula in the left pyriform sinus, which was cauterized using a diode laser (Figure 2A,B). After cauterization of the fistula, fibrin glue (BOLHEAL®, factor XIII with fibrinogen; Astellas, Japan) was applied to the cauterized surface of the same site to prevent subsequent PSF formation (Figure 2C). No relapse of PSF infection was observed 8 months after surgery (Figure 3).

Case 2: Another 3‐year‐old girl with left posterior cervical swelling and fever received an intravenous ceftriaxone antibiotic drip at the pediatric clinic. Two days later, swelling of the anterior cervical region was noted during ultrasonography, acute purulent thyroiditis was identified during contrast‐enhanced CT, and ampicillin/sulbactam was infused to improve the symptoms. Subsequent contrast‐enhanced CT and esophagography revealed a left‐sided PSF. A laryngoscope was inserted under general anesthesia, and after the PSF was confirmed, it was cauterized using a CO2 laser. Fibrin glue (Beriplast P®, factor XIII with fibrinogen; CSL Behring K. K., Australia) was applied to the same site to close the fistula. No subsequent recurrence was observed 3 years after surgery.

## 3 DISCUSSION

PSF was first reported by Tucker et al.^5^ in 1973 as a cause of anterior cervical abscesses. PSF is a congenital entity formed due to the abnormal development of the third or fourth branchial cleft.^6^ PSF begins at the pyriform fossa, passes through the cricothyroid muscle, and terminates laterally in the thyroid gland, which can cause cervical abscesses and acute purulent thyroiditis.^7^ In the past, open surgery of the fistula and related hemithyroidectomy were performed, but recently, there have been reports of endoscopically cauterizing the fistula using a laser.^8^ There are also reports showing the effectiveness of endoscopic coblation as a first‐line treatment for PSF.^9^ There is still a risk of fistula recurrence if only its surface is cauterized.^10^ A method of preventing recurrence by absorbing suture after cauterizing a pear or fistula with a laser was presented.^11^ Recently, there have been reports of covering the ablated surface of the fistula with fibrin glue via a laser,^4^, ^12^ and we performed the same procedure in this presented cases. In both cases, the wound was covered with fibrin glue after cauterization of the fistula using a diode or CO_2_ laser, and no recurrence has been observed thus far. Regarding the laser used, the use of potassium‐titanyl‐phosphate, CO_2_, and diode lasers has been reported.^3^, ^4^, ^13^ In children, open surgery or hemithyroidectomy are invasive treatments that impair aesthetics, and the alternative of fistula ablation using an endoscope is very effective. In the past, microscopes were often used in otorhinolaryngological surgery, but in recent years, various endoscopic surgeries have been introduced in otorhinolaryngology.^14^ In this study, no recurrence was observed after surgery for PSF using fibrin glue after laser ablation, which can therefore, be considered an effective method. However, it is necessary to observe whether there is a recurrence in the future.

## 4 CONCLUSION

We report two cases in which PSF was closed endoscopically using laser ablation and fibrin glue was used to prevent recurrence. Different types of lasers and fibrin glue were used in both cases, and no postoperative recurrence was observed in either case. Previously, invasive open surgery was performed to treat PSF. In contrast, the above procedure is not physically invasive and can become the surgical method necessary for aesthetic effectiveness.

## AUTHOR CONTRIBUTIONS

Dr. Manami Momii and Dr. Toshiaki Kawano, the lead author, wrote the manuscript, performed a literature review, and edited the manuscript. Dr. Sonoka Takakura and Dr. Kaori Tateyama obtained patient perspectives and consent and summarized the clinical case notes. Dr. Takashi Hirano edited the final script and contributed to the discussion and key messages. Dr. Masashi Suzuki suggested the case report and approved the final manuscript.

## FUNDING INFORMATION

The authors have no funding, financial relationships.

## CONFLICTS OF INTEREST

The authors have no conflicts of interest to disclose.

## CONSENT

Written informed consent was obtained from the patient to publish this report in accordance with the journal's patient consent policy. If the patient/participant is unable to give written consent or deceased, the same can be obtained from the patient's next of kin. If the patient is a child or under the age of 16, written consent should be obtained from a parent/guardian.

## PROOFS

Please refer to attachment.

## Data Availability

None.
